# How Financial and Reputational Incentives Can Be Used to Improve Medical Care

**DOI:** 10.1111/1475-6773.12419

**Published:** 2015-11-17

**Authors:** Martin Roland, R. Adams Dudley

**Affiliations:** ^1^ Department of Public Health and Primary Care Institute of Public Health University of Cambridge Robinson Way Cambridge CB2 0SR UK; ^2^ Center for Healthcare Value Philip R. Lee Institute for Health Policy Studies University of California, San Francisco San Francisco CA

**Keywords:** Incentives in health care, quality improvement, report cards, quality of care

## Abstract

**Objectives:**

Narrative review of the impact of pay‐for‐performance (P4P) and public reporting (PR) on health care outcomes, including spillover effects and impact on disparities.

**Principal Findings:**

The impact of P4P and PR is dependent on the underlying payment system (fee‐for‐service, salary, capitation) into which these schemes are introduced. Both have the potential to improve care, but they can also have substantial unintended consequences. Evidence from the behavioral economics literature suggests that individual physicians will vary in how they respond to incentives. We also discuss issues to be considered when including patient‐reported outcome measures (PROMs) or patient‐reported experience measures into P4P and PR schemes.

**Conclusion:**

We provide guidance to payers and policy makers on the design of P4P and PR programs so as to maximize their benefits and minimize their unintended consequences. These include involving clinicians in the design of the program, taking into account the payment system into which new incentives are introduced, designing the structure of reward programs to maximize the likelihood of intended outcomes and minimize the likelihood of unintended consequences, designing schemes that minimize the risk of increasing disparities, providing stability of incentives over some years, and including outcomes that are relevant to patients' priorities. In addition, because of the limitations of PR and P4P as effective interventions in their own right, it is important that they are combined with other policies and interventions intended to improve quality to maximize their likely impact.

There has been much policy discussion over the last decade about redesigning health care payment strategies to create alignment between the goals of clinicians and payers and, by extension, patients and society. This conversation has been stimulated in part by a growing body of research addressing the responses of clinicians to the three traditional approaches to payment—fee‐for‐service, capitation, and salary—and the impact of adding incentives such as pay‐for‐performance (P4P) and public reporting (PR) to these primary payment systems. The other stimulus to the conversation is the implementation of new payment programs that reflect the evolving consensus that each of the primary payment systems has the potential for important unintended consequences.

The goal of this paper was to help focus those policy discussions on key issues that have received less attention in both the literature and in the programs implemented. Building on the extant literature, in Section I, we first define our use of certain terms and provide a very brief summary of the main findings about P4P and PR to date, including spillover effects and the impact of incentives on disparities. In Section II, we describe some novel approaches to understanding incentives from the behavioral economics literature. In Section III, we offer a research agenda that addresses issues not yet well studied but essential to successful incentive program implementation. In doing this, we start from the theoretical basis offered earlier in this issue of *Health Services Research* by Conrad (2015) and ask which aspects of those conceptual models still need empirical support after assessment of the literature in Section II. Section IV recognizes that, while we still have incomplete empirical support for our theoretical models, decisions must be made now about design of incentive programs, and in this section we therefore offer recommendations to policy makers.

In keeping with Conrad (2015), we limit our discussion to incentives targeting either physicians or hospitals.

## Section I: Brief Summary of the Literature

In this section, we first define some terms related to incentive systems used in health care. Next we briefly summarize the literature.

Historically, there have been three strategies used to pay for medical care: (1) fee‐for‐service (FFS), (2) salary, and (3) capitation (CAP, or fixed payments per patient per unit time, sometimes also referred to as global payments). Each of these fundamental approaches creates very different payment incentives for providers. In Table 1, we list both the intended benefits and potential unintended consequences of each payment strategy.

**Table 1:** Advantages and Disadvantages of Different Payment Systems

Type of Model	Description	Potential Advantages	Potential Disadvantages
Fee‐for‐service	Physician gets paid for each service provided	As providers get paid for each new service, they may be able to increase services available as demand grows May stimulate high quality if there is competition based on performance Simple to administer and enforce once established	Overprovision of services No incentive for team‐based care No incentive to consider overall health care costs Encourages short doctor visits No change in rewards based on experience or quality
Salary	Payment of annual salary to work a certain number of hours per week	Contains costs unless associated with unnecessary referral or test ordering Simple to administer and enforce Reduced incentive to overtreat compared to fee‐for‐service	Can mean low productivity or quality of service No incentive to see more patients or provide better access
Capitation	Payment made for every patient enrolled in the doctor's practice	Contains costs Defined patient population facilitates preventive care Incentive to keep costs per patient low Encourages population coverage by incentivizing physicians to take on more patients	Complex to establish and enforce Can mean low quality, particularly through underprovision of care May encourage doctors to select the healthiest patients

*Source*: Adapted from Conference Board of Canada (2014).

It has become clear that the unintended consequences listed in Table 1 all occur with regularity. For this reason, in the past two decades, there has been increasing interest in adding new incentives to each of these three payment contexts (Jha et al. 2003; Rosenthal et al. 2005; Bardach et al. 2013). In particular, both financial incentives to improve performance (pay‐for‐performance, or P4P) and reputational incentives (public reporting, or PR) have been widely implemented.

One of the key questions asked in the P4P and PR literature is, “Do they work?” In our view, this is the wrong question, as P4P and PR—like the three primary payment strategies—can have both beneficial intended outcomes and unintended perverse outcomes. We now briefly review what is known about P4P and PR.

### The Pay‐for‐Performance Literature

In terms of P4P, excellent systematic reviews and other detailed summaries of the literature exist elsewhere (Conrad and Perry 2009; Van Herck et al. 2010; Eijkenaar et al. 2013; Damberg et al. 2014), and Conrad provides a thorough linkage of the research findings to theories of incentives earlier in this issue (Conrad 2015).

In policy discussions, we have often heard the findings of these reviews described as “the results are mixed,” but this is incomplete. While there are some studies showing that P4P improves performance as expected and others show no effect, we are not aware of any studies that found P4P caused statistically significant reductions in the targeted performance measures. Therefore, the literature is most aptly summarized as suggesting P4P generally creates some stimulus to improve, but that effect can be mitigated or even overwhelmed by other factors, most likely the incentives of the primary payment mechanisms onto which P4P has been grafted. The implication of this for policy makers is that the magnitude of the response to a given incentive is uncertain and likely will vary by the context into which it is applied (including the underlying fee‐for‐service, salaried, or capitated system).

An issue about which P4P literature is more truly “mixed” in the sense of having some positive and some negative findings is the impact of incentives on vulnerable patients and the providers who care for them. Almost all research shows that such providers start at lower performance levels than providers serving the general population. P4P payments based on absolute performance would be lower to safety net providers and, by extension, P4P could harm vulnerable patients (Alshamsan et al. 2010). In addition, it is possible that, within a provider's population, he or she might focus on more affluent, educated patients, if these were judged to be the patients for whom it is easiest to receive additional remuneration (Victora et al. 2000). However, there also are reports of safety net providers reducing performance gaps over time (Baker and Middleton 2003; Doran et al. 2008a; Werner, Goldman, and Dudley 2008), suggesting that incentives might benefit vulnerable populations.

There can be other unintended consequences. “Cream‐skimming,” or disenrollment of high‐risk or noncompliant patients, has been reported (McDonald and Roland 2009; Chang, Lin, and Aron 2012). Gaming of the performance measures has also occurred, including selective exclusion of patients on whom the physician has failed to secure maximum remuneration in the previous year (Gravelle, Sutton, and Ma 2010) or increasing exception reporting (in which physicians are allowed to choose themselves whether they report certain patients for performance measurement) (Kontopantelis et al. 2012). Although the economic literature suggests that individuals may be more sensitive to incentives when the result is a penalty rather than a reward (Kahneman and Tversky 1998), there is also some evidence that penalties may be more likely to produce unintended consequences, especially if the physicians or hospital leaders feel the measures are out of their control or the penalty is unfair in some other way (Morreim 1991; Werner et al. 2002).

On the other hand, there are some positive lessons from the literature. In particular, absolute payments (simply paying for each instance of high performance) usually create more effective incentives than tournaments among providers or other schemes that pay on a relative basis, for example, rewarding the top 25 percent of providers (Conrad and Perry 2009; Van Herck et al. 2010; Eijkenaar et al. 2013; Damberg et al. 2014).

### The Public Reporting Literature

There have been no randomized controlled trials of the impact of PR on performance. Before‐and‐after design studies are often complicated by the fact that PR is almost always implemented with a lag between the announcement of what measures will be included and actual reporting, so providers can start improving their performance before the program begins. Furthermore, PR is often introduced with other system changes (such as the introduction of financial incentives or the establishment of a learning collaborative) that might have as much or more impact on performance as PR. Examples of this phenomenon include the improvement in CAHPS Hospital Survey in Aligning Forces for Quality communities, in which PR was only one component of multi‐stakeholder efforts to improve performance (Shaller and Zema 2014).

However, we do have evidence from a natural experiment in Wisconsin that has been closely studied. In the Madison region, a business alliance published and widely disseminated a hospital performance report that was based on information from a state database. Hospitals in the rest of Wisconsin had the same performance information in the database, but it did not face PR. Hibbard et al. surveyed hospitals and consumers before and after the first PR and continued to collect the performance information from the state database. They found that hospitals in the part of the state that had PR engaged in more quality improvement activities (Hibbard, Stockard, and Tusler 2003) and improved more over time, and that PR affects hospitals' reputations with consumers (Hibbard, Stockard, and Tusler 2005). In addition, the introduction of PR has been associated with the elimination of long‐standing quality issues. For example, the volume–outcome relationship previously observed for coronary artery bypass grafting in California disappeared after the introduction of PR, without any formal consolidation of care in regional centers (Marcin et al. 2007). Studies like this suggest that PR does provide a meaningful stimulus, but the absence of randomized trials means that the magnitude of that stimulus or its ability to balance the incentives created by the primary payment system remains unknown.

As with any incentive system, PR can have unintended consequences. For instance, there is evidence that New York cardiac surgeons became more reluctant to operate on black and Hispanic patients following the introduction of PR (Werner 2005).

Some of the advantages and disadvantages of P4P and PR are summarized in Table 2. While some of the effects appear similar, the mechanisms can potentially be quite different. For example, P4P may widen disparities by financial rewarding those who care for healthier, wealthier patients, whereas PR may widen disparities by encouraging wealthier patients to seek better quality providers.

**Table 2:** Potential Advantages and Disadvantages of P4P and PR

Type of Model	Description	Potential Advantages	Potential Disadvantages
Pay‐for‐performance (P4P)	Pay physicians (or a health care team) based on achieving quality or volume targets	Financially rewards high‐quality health care Demonstrates commitment to evidence‐based health care Transparent rewards process (at least to the providers, sometimes also to patients) Can be used to focus attention on underserved or high‐risk groups	Can distract providers from caring for nontargeted conditions No consensus about optimal program strategy Complex to set up, find, and agree on evidence‐based quality measures Effectiveness and cost‐effectiveness unclear Difficult to measure outcomes in complex cases Has the potential to widen disparities
Public reporting (PR)	Release data about clinical performance to the public	Can improve reputation, leading to both psychological rewards and/or financial benefit (either through more patient referrals or being able to command a higher price) Demonstrates commitment to evidence‐based health care Transparent rewards process Can be used to focus attention on underserved or high‐risk groups	Can distract providers from caring for nontargeted conditions Difficult to display a range of measures accessible to a lay public Effectiveness unclear Difficult to measure outcomes in complex cases Has the potential to widen disparities Uncertainty over whether the public or providers are the actual target audience

## Section II: Novel Research that Informs the Policy Debate

As it is clear that no existing incentive system is yet optimal, new research is needed. Here, we highlight some important additions to the literature. To inform the previous section, we relied on the cataloging of the literature performed by Damberg et al. (2014) in a recent report for the U.S. Department of Health and Human Services in which we participated, the review by Conrad in this issue (2015), recommendations of the expert team assembled for this project, and our own readings of the literature in the months since the Damberg and Conrad reviews. In this section, we address the core question of whether programs are likely to have any effect at all including new evidence using novel study designs and new types of data that may be included in P4P schemes. We address the question of who the main winners and losers from incentive schemes are likely to be, including possible negative impacts on vulnerable populations. We also describe the potential for programs to have an effect beyond the disease or populations targeted (spillover effects) and some other features that are likely to predict successful implementation.

### Responses to Financial Incentives

New evidence from experimental economics represents interesting additions to the literature on financial incentives. The general design of these experiments is that study subjects are asked to consider hypothetical patients of varying health status and utilization needs, and they are told the optimal level of utilization (from a patient benefit standpoint). The study subjects then are asked to decide how many services to deliver. In the fee‐for‐service condition, study subjects receive more payment for deciding to deliver more services; in the capitation condition, they receive less for deciding to deliver more services. To create a real tradeoff between the study subjects' income and patient benefit, subjects are told either (1) that a donation will be made to a health care charity and that these donations will be decreased for each instance in which they did not provide the optimal level of utilization, or that (2) variations from optimal utilization will cause lower payments to other “patient” study subjects (who are also students). In the example of donation to charity, study subjects also observe the actual donation, so they know it is a real benefit to patients that can be decreased by their behavior.

Using protocols like these, Hennig‐Schmidt, Selten, and Wiesen (2011) and Green (2013) both find that medical and economics students, respectively, respond to fee‐for‐service and capitation as expected, with overuse in the former and underuse in the latter, to the detriment of patients in both studies. Keser, Peterle, and Schnitzler (2014) also found that fee‐for‐service payments led medical students to overuse, but that P4P tied to providing the optimal level of services (from the patient perspective) could mitigate this. However, although the expected responses to incentives were found generally, the effects were mitigated substantially by patient characteristics: sicker patients got more services than healthy patients under any payment scheme in all three studies.

In addition, Brosig‐Koch et al. (2015) assessed the differences in responses to fee‐for‐service and capitation in three groups of study subjects asked to make the similar income‐versus‐patient benefit tradeoffs. The first two groups, medical and nonmedical students, were studied in a lab environment. The third group, practicing physicians, were studied in their offices and offered fourfold larger payments than the students. Income‐seeking behavior in response to fee‐for‐service or capitation incentives—at the risk of reduced contributions to a charity health care provider—was lower among medical students than among nonmedical students and much lower among physicians. Plausible explanations for medical students being more likely to forego income for patient benefit relative to nonmedical students include either a selection effect—with people choosing to go to medical school having more altruism than average—or the impact of clinical knowledge or professional socialization on clinical behavior. There are other possible explanations for the difference between physicians and medical students. These include that physicians are further out on their marginal utility of income curve, so that even fourfold larger payments do not provide as much utility to them as the smaller payments to students. Other plausible hypotheses include that the impact of additional knowledge or socialization during residency and practice or a social acceptability response bias that grows with time spent in clinical work reduces physician responses to financial incentives in experimental situations. These hypotheses need to be tested empirically.

If these findings reflect anything other than a social acceptability response bias, however, they are important for policy makers. Physicians often argue in policy discussions that they put patients first and do not need to be incentivized. These data would suggest that this is at least partly true, and also not completely true.

### Variation among Individuals in Response to Incentives

In an extension of the Hennig‐Schmidt study, Godager and Wiesen (2013) analyzed the heterogeneity of the responses of the study subjects to the fee‐for‐service and capitation incentives. They found statistically, clinically, and financially significant variation among medical students in the extent to which their behavior reflected the opportunity to increase income versus the opportunity to provide health benefits to patients. This suggests that altruism varies substantially among physicians in training.

This is extremely important for policy makers to understand. If provider altruism and the weights individual providers put on income and patient benefit vary widely, then it will be very difficult to design an incentive system that aligns all providers' goals with patients' and society's goals.

### Effect of Paying More for What Is Harder to Do

P4P programs need not make the same payment for all patients, and there are strong reasons not to do so. Chief among these are that a P4P performance target may be more difficult to achieve in some patients than in others or is of greater value when achieved in one patient than another. For example, while blood pressure control is important and valuable for all patients, it is harder to achieve among patients with chronic kidney disease. Therefore, for a clinician to achieve blood pressure control in such patients requires more work. If the P4P program does not incorporate this extra work into its payment scheme, this increases the risk of unintended consequences, such as clinicians wanting to avoid having patients with kidney disease in their panels. For diabetics, achieving blood pressure control is more valuable for society than controlling blood pressure in a nondiabetic because diabetics are at higher risk of devastating and expensive vascular events like strokes and myocardial infarction if their blood pressure is not controlled. Paying more for blood pressure control among diabetics, then, is an efficient use of P4P resources.

### Spillover Effects

Damberg et al. (2014) report that both negative and positive spillover effects have been documented. Negative spillover effects may result from relative neglect of unincentivized conditions. There is some evidence from the United Kingdom's P4P program that improvements in quality for incentivized indicators were at the expense of some detriment in quality for unincentivized conditions (Doran et al. 2008a). On the positive side, Kristensen et al. (2014) found some evidence that when the initial effect of reduced hospital mortality from the introduction of an HQID program in the United Kingdom was lost in the longer term, this might have been the result of positive spillover effects into nonincentivized conditions. The possibility of negative spillover effects is particularly important because P4P often targets aspects of care that can be measured relatively easily (e.g., blood sugar control in diabetes) and therefore risks neglecting aspects of medical care in which processes and outcomes may be more difficult to measure (e.g., mental health care).

### The Emergence of Patient‐Reported Information

Later in this issue, Schlesinger, Grob, and Shaller discuss patient‐reported information in detail (2015). The generation of such information has advanced rapidly over the last decade. Sentinel events include the national implementation of PR of the CAHPS Hospital Survey in the United States in 2008, the widespread use of patient surveys in the United Kingdom, and the U.S. National Institutes of Health's commitment to the Patient Reported Outcomes Measurement Information System.

It is important to distinguish between patient‐reported outcome measures (PROMs) that include health status measures and clinical outcomes and patient‐reported experience measures, which are commonly based on patient surveys. PROMs are only suitable for P4P or PR where the outcome measured is under the control of the person or institution being incentivized. It would be inappropriate, for example, to incentivize a primary care physician on the Kidney Disease Quality of Life (KDQOL) scale among his patients—this is influenced by too many factors outside the control of the primary care team. However, Medicare requires U.S. nephrologists to collect the KDQOL annually on all dialysis patients, so this PROM would be both relevant to nephrologist's practice and available for P4P and PR. Similarly, for most surgeries, hospitals and surgical teams play a major role in determining surgical outcomes and so these are potentially more suitable for use in PR or P4P. The NHS in England now publicly reports change in health status for all patients in the NHS undergoing hip and knee surgery, hernia, and varicose vein surgery, though the evidence to date is this has had little impact on outcomes (Varagunam et al. 2014).

Measures of patient experience also are substantially under the control of the provider and are widely used in P4P and PR. Patient experience is an important dimension of quality in its own right and is important to include alongside clinical measures of quality because of evidence that financial incentives targeting defined clinical tasks may reduce the patient‐centeredness of consultations (Gillam, Siriwardena, and Steel 2012). However, linking pay to survey results is complex because of low response rates in surveys and the difficulty of making survey results sufficiently reliable to be a basis for payment (Roland et al. 2009). This contributed to the decision to abandon an experiment in the United Kingdom to link physician pay to patient experience survey results after a short period.

There is continued interest in finding new methods of incorporating patient feedback into public reporting systems. One potentially useful source of information from patients are free‐text narrative reviews, which, for example, the NHS in the United Kingdom began collecting as part of “NHS Choices” in 2007 (www.nhs.uk). A recent review of narrative patient feedback from a German website found that patient comments were mostly positive but occasionally highlighted important shortcomings ranging from wait times to physician competence and cost (Emmert et al. 2014). However, these voluntary reports are also subject to response bias, and it remains unknown how to collect and moderate the comments and how to incorporate them into P4P and PR systems that are otherwise entirely quantitative (Greaves, Millett, and Nuki 2014). A further issue is whether making comments public would suppress patients' willingness to submit reviews about providers they planned to use in the future, either because they would not want to hurt the providers or because they feared retaliation (Schlesinger, Grob, and Shaller 2015).

### Targeting the Appropriate Entity (Individuals, Groups, Institutions, System)

Economic theory suggests that incentives are unlikely to work if the person doing the work sees no benefit to himself. The only randomized trial comparing incentives to individuals versus clinical groups bears this out: individually incentivized physicians were more likely to achieve blood pressure control or make the right medication change than physicians for which the P4P was paid to the group (Petersen et al. 2013).

Of course, it is possible that in other circumstances an individual would respond to an incentive to his team or the institution or system in which he works. Furthermore, it may at times be most appropriate to have team‐level incentives. For example, in the United Kingdom's implementation of HQID, rewards were initially given to clinical teams that could employ additional staff to improve care. There was no direct financial reward to the doctors or nurses involved, but they could see the results of their efforts in increased staffing and/or support. This motivation changed when the scheme was subsequently revised so that rewards became penalties.

Sometimes the incentive may need to be applied at a much higher level. For example, one of the current challenges in health care is providing integrated care for patients with multiple chronic conditions. Were incentives to be used to provide better integrated care, they would need to be addressed at promoting closer cooperation between health and social care organizations. A second example might relate to teenage pregnancy. There would probably be limited value in incentivizing primary care physicians against the local teenage pregnancy rate, but the organization with responsibility for providing easily accessed family planning centers could potentially be incentivized against an outcome of teenage pregnancies.

Unfortunately, it is also possible to design an incentive system that does little to ensure that people doing the work will recognize any benefit from the incentive. For example, the Brazilian national primary care P4P program provides money to municipalities who in turn are responsible for providing primary care. However, there is nothing to stop municipalities diverting the rewards gained to other areas of need (e.g., education), thus reducing the incentive on primary care physicians to organize their primary care units to provide better care.

The key summary point is that the aspect of care being incentivized should be under the control of the person or organization being paid, and that person or organization should be able to see some benefit from achieving good performance.

### Impact of Uncertainty

The last 25 years have seen enormous change in how providers are paid and the introduction and evolution of transparency initiatives that impact their reputation. Furthermore, many trends have proved impermanent; for instance, in the 1990s it was widely suggested that provider payment in the United States would soon be primarily based on capitation. Many medical groups invested in developing the infrastructure to accept risk‐based contracting, only to see that payment method slowly recede. Several reported that uncertainty about both whether they could meet performance targets and what the financial rewards would be when finally paid made return on investment calculations difficult (Lipton et al. 2005).

It would not be surprising, then, if clinical organizations viewed the next change as temporary. This is critical, because if financial returns are uncertain, organizations discount the rewards and may be less willing to commit to and invest in change. This is also important in light of our growing understanding, stemming from the application of behavioral economics and prospect theory to health care, that individuals (and, by extension, the organizations they lead) may weight losses more heavily than gains. Uncertainty may increase the impact of loss aversion, as it reduces the certainty that losses can be avoided.

A good example of addressing long‐term considerations is the Alternative Quality Contract introduced by Blue Cross Blue Shield of Massachusetts. While this program includes description of performance metrics for the current year, it also has a 5‐year plan that allows providers to know whether and how performance measurement and payment will change over a longer time horizon, and it seems to have effectively stimulated improvement (Song et al. 2011).

### Effect of Involving Clinicians in Incentive System Design

Psychological theory suggests that providing external rewards may reduce internal motivation (Deci, Koestner, and Ryan 1999). This is true not just of P4P and PR, but of all the payment systems in Table 1. To date, there is little empirical evidence that this has happened with P4P and PR; one empirical study in the United Kingdom suggested that physicians' internal motivation was not damaged by the introduction of P4P (McDonald et al. 2007). Nonetheless, incentives that, by their design, align with professional values are clearly preferable to incentives that do not. However, it has proven difficult for payers and policy makers by themselves to anticipate when incentive systems might cause misalignment and unintended consequences.

Furthermore, while the physician community has been generally skeptical of P4P and PR, there is evidence that they are more comfortable with incentive programs when they are involved in the program planning and design. In a P4P program in New York, providers were invited in to help determine payment amounts and how those should vary by patient characteristics. Surveys of providers after a year under this system show that they understood how their performance was measured, accepted the clinical priorities established under the system, and felt that the performance measurement was accurate (Begum et al. 2013).

## Section III: Proposed Research Agenda

As we have discussed above, there is no simple answer to the question “Do P4P and PR work?” and the answer in an individual setting is highly dependent on the context and details of implementation. If P4P and PR were new drugs, we would want to know much more about them before considering using the new treatment—the conditions they helped, the optimum dose, side effects, how much they improved outcomes, adverse reactions, cost, and so on. In contrast, many payers in the past decades have turned uncritically to P4P and PR in their search for a quick cure for poor quality. What is now needed is a research agenda that addresses how and in what circumstances P4P and PR are effective and how unintended consequences may be avoided. These include the following:

*Impact of Incentives on Vulnerable Populations*

1. Given our current capacity to measure and reward quality, does offering incentives increase or decrease disparities in care? Are there strategies that could be adopted to ensure that creating incentives does not worsen care for the most vulnerable patients?
2. What is the best way of providing incentives for providers in underserved areas?

*Reputational versus Financial versus Regulatory Incentives*

1. How much and in what circumstances are reputational incentives important?
2. Can (and should) PR be separated from P4P?
3. Does adoption of measures for accreditation or licensing standards increase the impact of P4P and PR? Or does it result in ceiling effects that limit the impact of P4P and PR?

*Incentive Design*

1. How important is the size of the incentive?
2. What are the relative merits and drawbacks of rewards versus penalties?
3. How can quality be maintained if incentives are withdrawn once satisfactory levels of quality are achieved?
4. How important are spillover effects—how can positive ones be encouraged and negatives ones discouraged?
5. Should payers be encouraged to use a common set of metrics in P4P schemes? Or should indicators be adaptable to local circumstances and needs?
6. What differences in implementation of P4P and PR are needed depending on the payment scheme onto which they are being grafted (e.g., fee‐for‐service, salary, capitation). How does the development of accountable care organizations affect the development of P4P and PR?
7. How can clinical or policy priorities (such as paying more for reaching clinical goals that are harder to achieve or for reducing disparities) be incorporated into incentive payment levels?

*Responses to Incentives of Organizations and the Individuals within Them*

1. How can quality best be rewarded when it is dependent on the work of a team rather than an individual?
2. How can individual clinicians be motivated to support P4P and PR programs when they do not receive any personal benefit (e.g., when the bonus or quality rating goes to the hospital)?
3. Does changing incentive systems frequently decrease responsiveness of provider organizations to current incentives?

## Section IV: Recommendations for Policy Makers

In general, P4P and PR, when designed properly, appear to have some positive impact on quality of care, but neither is a magic bullet. Effects generally have been less than payers and policy makers had hoped for, so P4P and PR should always be seen as part of a wider quality and outcomes management strategy. Furthermore, unexpected consequences have been common, although we now know more about how to avoid them. Nevertheless, P4P and PR do have a place, partly because none of the primary payment systems create perfect incentives themselves. In fact, P4P and PR should be viewed as among a number of novel approaches that have been grouped under the term “value‐based purchasing” (Damberg et al. 2014). Other approaches, for example, include accountable care organizations and bundled payments—which seek to align incentives between providers, payers, and patients. P4P and PR may also form part of wider reforms to the organization and financing of medical care designed to improve the coordination and efficiency of care, such as the patient‐centered medical home (Korda and Eldridge 2011).

On the basis of what is already known, we make the following recommendations to policy makers:

*Fit P4P and PR to the Payment Context in Which They Are Introduced*

In the United States, most of the system seems to be headed to global or bundled payments. On their own, these approaches give clinicians incentives to do as little as possible for as many people as possible. This would suggest that P4P and PR could increase the alignment of the overall payment scheme with patients' and policy makers' goals if they were added to global payments (e.g., as received by an accountable care organization) and included measures of underuse and of patient‐reported outcomes. While these capitation‐based approaches are spreading, however, much of the U.S. system is still fee‐for‐service. Fee‐for‐service gives clinicians the incentive to provide as many services as possible, regardless of whether those services are necessary, are done right, or are consistent with patient preferences. Therefore, P4P and PR, when added to fee‐for‐service may need to focus in particular on indicators of overuse and where shared decision making needs to occur.

In some other parts of the U.S. system—such as the Department of Veterans Affairs, the Department of Defence, and some prison systems—most clinicians are paid by salary. By itself, this approach gives clinicians an incentive to do as little as possible for as few people as possible. Therefore, P4P or PR added to these systems should focus on measures of access as well as underuse and measures of patient experience.

*Allow Clinicians to Help Design the Program*

This will reduce the risk of conflicts between professional, financial, and managerial incentives that might increase the risk of unintended outcomes. While there is mounting evidence that physicians and clinical organizations respond to incentives, we also find the lab and field experimental data that medical students and physicians are more willing than others to give up income for the sake of patient benefit compelling. This suggests that they have substantial intrinsic motivation and commitment to their patients. This likely can be harnessed to optimize an incentive system. Furthermore, as data suggest altruism varies among individuals, clinicians will be better positioned than anyone else to explain their own likely responses to any proposed scheme.

*Ensure That P4P Payments Are Large Enough to Change Behavior*

We know on the whole that larger incentives are likely to have more impact than small ones (Mullen, Frank, and Rosenthal 2010; Werner et al. 2011; Roland and Campbell 2014). We also note that the United Kingdom has recently chosen to reduce the magnitude of P4P incentives from 25 to 15 percent of physicians' income. It is not possible to say what the “correct” level of P4P payments should be and this will be dependent on the underlying payment system and also what behavior is being incentivized. The payments should be made at a level that is likely to change behavior, but not so great as to increase the probability of the perverse or unintended consequences of P4P.

*P4P Should Be Paid Out for Each Patient in Which the Right Process or Outcome Is Achieved, Rather Than Based on Thresholds, Competitive Payments to Top‐Ranked Providers, or Other Complicated Formulae*

The only obstacle to implementing this is that it can be difficult a priori to know the exact budget. This, for instance, is a challenge when the Medicare program is required to adopt budget‐neutral policies. However, clearly major experimentation with payment is needed in Medicare, so options include allowing for uncertainty about budget neutrality or using large bonuses and setting payments so they are expected, when combined with the P4P payments, to generate slight savings.

*P4P and PR Should Include an Emphasis on Patient‐Reported Information*

There is growing use of patient experience scores in PR. However, this is only one type of patient‐reported information. Patient narratives and complaints are other sources of information that patients find compelling. However, as use of these is just beginning, any incorporation of them into P4P or PR should include a plan to monitor for changes in consumers' use of these (e.g., some consumers may be more reluctant to report complaints, especially about physicians or hospitals they may need to use in the future, if they know that they will become public). Patient‐reported outcomes measures are now available that are well validated and may be used in public reporting schemes. Their wider use in P4P should be limited to situations where the outcome is under the direct control of the physician or hospital being incentivized.

*Consider the Impact of the Scheme on Disparities*

There are several potential approaches to addressing this issue. One is to risk‐adjust measured performance for high‐risk patients or populations to reduce the chance of their being excluded or disadvantaged. A difficulty with this is that the provider is likely to have superior information about their patients than the information included in risk‐adjustment models (Dranove et al. 2003). All the situations in which cream‐skimming has been reported have involved risk‐adjusted measures, but the persistence of cream‐skimming suggests the providers did not believe the risk‐adjustment was adequate. This may be mitigated by post‐adjustment of payments using predefined patient or provider characteristics in order to reduce payment disparities (Damberg et al. 2015). An alternative approach adopted by the United Kingdom's Quality and Outcomes Framework is to allow physicians to exclude individual patients from single or groups of indicators (“exception reporting”) (Doran et al. 2008b). While this has led to concern that physicians would exclude those most in need, in practice only fewer than 5 percent of patients are excluded (Doran et al. 2012). The scheme reduces the chance that physicians may prescribe treatments that they judge not to be in the patient's best interest (e.g., rigorous cholesterol monitoring in a diabetic patient dying of lung cancer).

A particularly promising new approach would be to provide greater rewards specifically for achieving success with patients who are hard to treat. The rationale for this is consistent with incentive theory and does not rest on any political theory of equity: the P4P sponsor is simply paying more for what is harder to do. For example, in a program in New York City, P4P payments for achieving blood pressure control were doubled when patients had either low socioeconomic status or increased clinical complexity (Bardach et al. 2013).

*Use P4P Funds Efficiently*

A randomized trial has shown that P4P dollars can be focused in areas where increases in performance are either more difficult to achieve or offer more clinical benefit (Bardach et al. 2013). In a P4P system designed for maximally efficient use of P4P funds, policy makers would work with clinicians to identify these priority areas and then base P4P payment levels on the relative priorities.

*Offer a Multiyear Plan for P4P and PR*

To reduce uncertainty and facilitate planning by the providers that payers hope to incentivize, it is important for payers to commit to any new system for multiple years (at least three, preferably more). This will allow providers to calculate return on investment (for P4P) or estimate the impact of a PR program with greater certainty and may improve responses to incentives. As it is possible, however, that there may have been some design flaws in any program's first version, it may be helpful to also explicitly build in from the beginning periods for public comment and revision. By describing these in the initial multiyear plan, this approach offers some flexibility without reducing the appearance of long‐term commitment.

*P4P and PR in Relation to Other Quality Improvement Activities*

Both P4P and PR are also most likely to be effective when combined with other initiatives to help providers improve, such as quality improvement collaboratives or technical assistance, and neither P4P nor PR should be seen as stand‐alone interventions.

*Consider the Organizational System into Which P4P Is Being Introduced*

There are no trials that have differentiated between the type of provider entity (individual physicians, small practices, large medical groups or IPAs, individual hospitals, or combinations of these entities) in terms of their response to incentives. However, the response of any individual or organization will be influenced by the ease with which they can make improvements, and some differences in this domain can be expected by provider type. In particular, smaller groups tend to need more technical assistance, for example, with the implementation of an electronic records or learning how to measure performance and improve compared to their own prior performance, while larger groups tend to value access to regional benchmarks and to use consultants with clinical expertise to help them bring activities to scale.

*Monitor Continuously for Unintended Consequences after Implementation*

There are few incentive schemes that do not carry the risk of perverse or unintended consequences. These need to be anticipated. Evidence of varying degrees of altruism among physicians suggests that it will be impossible to design a payment system that aligns to the personal or professional values of all physicians. Potential adverse outcomes of all payment systems therefore need to be monitored.

*Address and Prevent Unintended Consequences through Sophisticated Design*

Including clinicians in designing the scheme will give them the opportunity to point out potential unintended consequences. In addition, where patients who are complicated—whether for social or clinical reasons—are at risk, clinicians are best‐positioned to answer questions about whether possible solutions are adequate. For example, in New York City's P4P program, clinicians helped the city determine “How much extra work do certain difficult patients represent compared to typical patients?” and responses to this question were used to determine the additional payments made for achieving good outcomes with these complex patients. Likely as a result of their early engagement, participating physicians responded in subsequent surveys that the measures were clinically meaningful and the payment scheme reasonable (Begum et al. 2013).

*Add New Measures to P4P and PR*

To date, many P4P and PR programs have adopted as performance indicators measures that were already in use by accreditation programs such as the Joint Commission for hospitals and HEDIS (Healthcare Effectiveness Data and Information Set) for physicians. This may in part explain the limited impact sometimes reported for incentive programs: by the time the measures are incentivized, clinicians have been working on them for years, and the range of performance has been reduced, making it more difficult to find a response to the incentive. Clearly, in the U.S. health care system, by the time measures have been the subject of accreditation reviews plus P4P and PR, national levels of performance far exceed what is observed for measures that have not received such focus. As there is now fairly strong evidence that providers respond to P4P and PR (it is the strength of the response that remains an open question), it is likely that policy makers could increase the rate of improvement across the system if they begin to adopt more novel measures in incentive systems. Examples of novel and clinically significant measures to be considered would include PROMs that meet the criterion of representing outcomes that are under the control of the person or institution being incentivized.

In the United States, there are excellent sources for novel measures that have been carefully validated and could be adopted. The National Quality Forum (NQF) serves the function of convening stakeholders for vetting measures, so NQF‐endorsed measures have already been reviewed and largely accepted. In addition, the NQF manages the Measures Application Partnership (MAP), which guides the U.S. Department of Health and Human Services on use of performance measures in P4P and PR. Thus, selecting measures consistent with NQF and MAP recommendations would ensure that new measures introduced to P4P and PR were already aligned with national initiatives.

In conclusion, the available literature suggests that providers respond to incentives, but the strength of the response and the frequency of unintended consequences both depend on the context in which incentives are introduced and the design of the incentive program. We also have a sense of how big financial incentives should be. Much has been learned about what unintended consequences are likely, and methods of reducing them are available. In addition, it is clear how to adjust the focus of P4P and PR to the larger payment context so that the systems combine to align overall payment with clinicians' and patients' goals. In the United States, it is likely that larger incentives, applied with more attention to how to help providers respond, will be needed to increase quality and improve outcomes rapidly.

## Supporting information

Appendix SA1: Author Matrix.
